# Supplementary material for: Effects of SARS-CoV-2 Omicron BA.1 Spike Mutations on T-Cell Epitopes in Mice
Source: Viruses. 2023 Mar 16;15(3):763. doi: 10.3390/v15030763 (PMC10056712; doi:10.3390/v15030763)

Supplementary Materials

Figure S1. Arrangement of two-dimensional peptide matrix. Peptide pools NTD-1~NTD-18 **(A)**, RBD-1~RBD-16 **(B)**, S1-1~S1-12 **(C)**, S2-1~S2-24 **(D)** in WT Spike, and peptide pools NTD-1~NTD-18 **(E)**, RBD-1~RBD-16 **(F)**, S1-1~S1-12 **(G)**, S2-1~S2-24 **(H)** in Omicron Spike were shown.

A

	NTD-10	NTD-11	NTD-12	NTD-13	NTD-14	NTD-15	NTD-16	NTD-17	NTD-18
NTD-1	1	2	3	4	5	6	7	8	9
NTD-2	10	11	12	13	14	15	16	17	18
NTD-3	19	20	21	22	23	24	25	26	27
NTD-4	28	29	30	31	32	33	34	35	36
NTD-5	37	38	39	40	41	42	43	44	45
NTD-6	46	47	48	49	50	51	52	53	54
NTD-7	55	56	57	58	59	60	61	62	63
NTD-8	64	65	66	67	68	69	70	71	72
NTD-9	73								

B

	RBD-9	RBD-10	RBD-11	RBD-12	RBD-13	RBD-14	RBD-15	RBD-16
RBD-1	74	75	76	77	78	79	80	81
RBD-2	82	83	84	85	86	87	88	89
RBD-3	90	91	92	93	94	95	96	97
RBD-4	98	99	100	101	102	103	104	105
RBD-5	106	107	108	109	110	111	112	113
RBD-6	114	115	116	117	118	119	120	121
RBD-7	122	123	124	125	126	127	128	129
RBD-8	130	131	132					

C

	S1-7	S1-8	S1-9	S1-10	S1-11	S1-12
S1-1	133	134	135	136	137	138
S1-2	139	140	141	142	143	144
S1-3	145	146	147	148	149	150
S1-4	151	152	153	154	155	156
S1-5	157	158	159	160	161	162
S1-6	163	164	165	166	167	168

D

	S2-13	S2-14	S2-15	S2-16	S2-17	S2-18	S2-19	S2-20	S2-21	S2-22	S2-23	S2-24
S2-1	169	170	171	172	173	174	175	176	177	178	179	180
S2-2	181	182	183	184	185	186	187	188	189	190	191	192
S2-3	193	194	195	196	197	198	199	200	201	202	203	204
S2-4	205	206	207	208	209	210	211	212	213	214	215	216
S2-5	217	218	219	220	221	222	223	224	225	226	227	228
S2-6	229	230	231	232	233	234	235	236	237	238	239	240
S2-7	241	242	243	244	245	246	247	248	249	250	251	252
S2-8	253	254	255	256	257	258	259	260	261	262	263	264
S2-9	265	266	267	268	269	270	271	272	273	274	275	276
S2-10	277	278	279	280	283	284	285	286	287	288	289	290
S2-11	291	292	293	294	295	296	297	298	299	300	301	302
S2-12	303	304	309	310	311	312	313	314	315	316		

E

	NTD-10	NTD-11	NTD-12	NTD-13	NTD-14	NTD-15	NTD-16	NTD-17	NTD-18
NTD-1	1	2	3	4	5	6	7	8	
NTD-2	9	10	11	12	13	14	15	16	
NTD-3	17	18	19	20	21	22	23	24	
NTD-4	25	26	27	28	29	30	31	32	
NTD-5	33	34	35	36	37	38	39	40	
NTD-6	41	42	43	44	45	46	47	48	
NTD-7	49	50	51	52	53	54	55	56	
NTD-8	57	58	59	60	61	62	63	64	
NTD-9	65	66	67	68	69	70	71	72	73

F

	RBD-9	RBD-10	RBD-11	RBD-12	RBD-13	RBD-14	RBD-15	RBD-16
RBD-1	74	75	76	77	78	79	80	81
RBD-2	82	83	84	85	86	87	88	89
RBD-3	90	91	92	93	94	95	96	97
RBD-4	98	99	100	101	102	103	104	105
RBD-5	106	107	108	109	110	111	112	113
RBD-6	114	115	116	117	118	119	120	121
RBD-7	122	123	124	125	126	127	128	129
RBD-8	130	131	132					

G

	S1-7	S1-8	S1-9	S1-10	S1-11	S1-12
S1-1	133	134	135	136	137	138
S1-2	139	140	141	142	143	144
S1-3	145	146	147	148	149	150
S1-4	151	152	153	154	155	156
S1-5	157	158	159	160	161	162
S1-6	163	164	165	166	167	168

H

	S2-13	S2-14	S2-15	S2-16	S2-17	S2-18	S2-19	S2-20	S2-21	S2-22	S2-23	S2-24
S2-1	169	170	171	172	173	174	175	176	177	178	179	180
S2-2	181	182	183	184	185	186	187	188	189	190	191	192
S2-3	193	194	195	196	197	198	199	200	201	202	203	204
S2-4	205	206	207	208	209	210	211	212	213	214	215	216
S2-5	217	218	219	220	221	222	223	224	225	226	227	228
S2-6	229	230	231	232	233	234	235	236	237	238	239	240
S2-7	241	242	243	244	245	246	247	248	249	250	251	252
S2-8	253	254	255	256	257	258	259	260	261	262	263	264
S2-9	265	266	267	268	269	270	271	272	273	274	275	276
S2-10	277	278	279	280	283	284	285	286	287	288	289	290
S2-11	291	292	293	294	295	296	297	298	299	300	301	302
S2-12	303	304	309	310	311	312	313	314	315			

Figure S2. Mapping of WT Spike specific T cell epitopes in BALB/c mice. BALB/c mice (n=6 per group) were injected with 5×10^8 VP ofAd5-Spike-BA.1. At 2 weeks after vaccination, the splenocytes were prepared, stimulated with peptide pools of WT NTD **(A)**, RBD **(B)**, S1 **(C)**, S2 **(D)**, and the T cell responses were measured by IFN-γ ELISpot assay. The two-dimensional peptide matrixes were used (as seen in Figure S1), with SFCs > 50 per 10^6 cells was considered as a positive peptide pool, and the candidate peptides were localized within the peptide matrixes. The results were expressed as mean ± SEM.

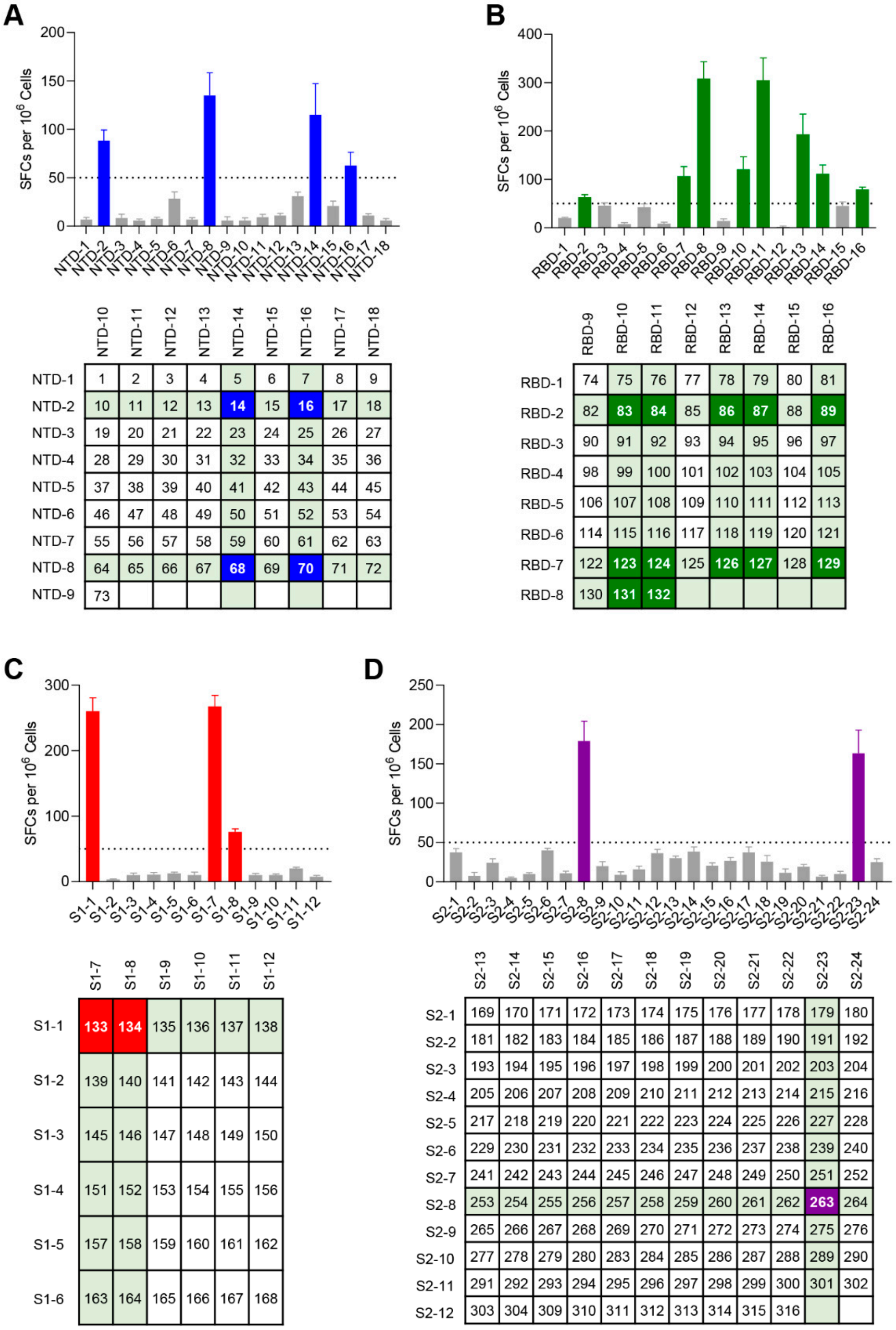

Figure S3. Mapping of Omicron BA.1 Spike specific T cell epitopes in BALB/c mice. BALB/c mice (n=6 per group) were injected with 5×10^8 VP of Ad5-Spike-BA.1. At 2 weeks after vaccination, the splenocytes were prepared, stimulated with peptide pools of Omicron NTD **(A)**, RBD **(B)**, S1 **(C)**, S2 **(D)**, and the T cell responses were measured by IFN-γ ELISpot assay. The two-dimensional peptide matrixes were used (as seen in Figure S1), with SFCs > 50 per 10^6 cells was considered as a positive peptide pool, and the candidate peptides were localized within the peptide matrixes. The results were expressed as mean ± SEM.

A

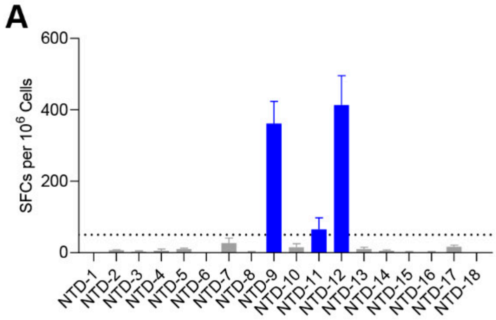

	NTD-10	NTD-11	NTD-12	NTD-13	NTD-14	NTD-15	NTD-16	NTD-17	NTD-18
NTD-1	1	2	3	4	5	6	7	8	
NTD-2	9	10	11	12	13	14	15	16	
NTD-3	17	18	19	20	21	22	23	24	
NTD-4	25	26	27	28	29	30	31	32	
NTD-5	33	34	35	36	37	38	39	40	
NTD-6	41	42	43	44	45	46	47	48	
NTD-7	49	50	51	52	53	54	55	56	
NTD-8	57	58	59	60	61	62	63	64	
NTD-9	65	66	67	68	69	70	71	72	73

B

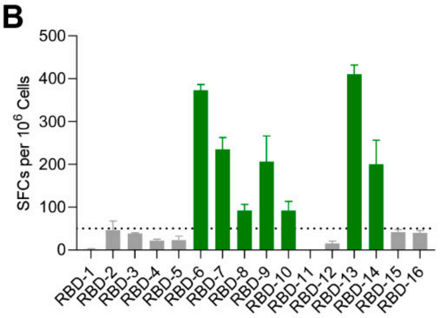

	RBD-9	RBD-10	RBD-11	RBD-12	RBD-13	RBD-14	RBD-15	RBD-16
RBD-1	74	75	76	77	78	79	80	81
RBD-2	82	83	84	85	86	87	88	89
RBD-3	90	91	92	93	94	95	96	97
RBD-4	98	99	100	101	102	103	104	105
RBD-5	106	107	108	109	110	111	112	113
RBD-6	114	115	116	117	118	119	120	121
RBD-7	122	123	124	125	126	127	128	129
RBD-8	130	131	132					

C

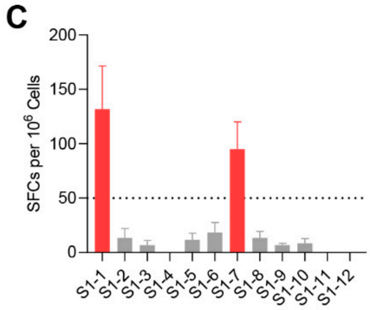

	S1-7	S1-8	S1-9	S1-10	S1-11	S1-12
S1-1	133	134	135	136	137	138
S1-2	139	140	141	142	143	144
S1-3	145	146	147	148	149	150
S1-4	151	152	153	154	155	156
S1-5	157	158	159	160	161	162
S1-6	163	164	165	166	167	168

D

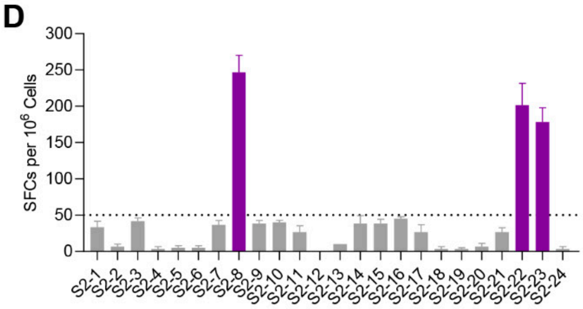

	S2-13	S2-14	S2-15	S2-16	S2-17	S2-18	S2-19	S2-20	S2-21	S2-22	S2-23	S2-24
S2-1	169	170	171	172	173	174	175	176	177	178	179	180
S2-2	181	182	183	184	185	186	187	188	189	190	191	192
S2-3	193	194	195	196	197	198	199	200	201	202	203	204
S2-4	205	206	207	208	209	210	211	212	213	214	215	216
S2-5	217	218	219	220	221	222	223	224	225	226	227	228
S2-6	229	230	231	232	233	234	235	236	237	238	239	240
S2-7	241	242	243	244	245	246	247	248	249	250	251	252
S2-8	253	254	255	256	257	258	259	260	261	262	263	264
S2-9	265	266	267	268	269	270	271	272	273	274	275	276
S2-10	277	278	279	280	283	284	285	286	287	288	289	290
S2-11	291	292	293	294	295	296	297	298	299	300	301	302
S2-12	303	304	309	310	311	312	313	314	315			

Figure S4. Mapping of WT Spike specific T cell epitopes in C57BL/6 mice. C57BL/6 mice (n=6 per group) were injected with 5 × 10^8 VP of Ad5-Spike-BA.1. At 2 weeks after vaccination, the splenocytes were prepared, stimulated with peptide pools of WT NTD **(A)**, RBD **(B)**, S1 **(C)**, S2 **(D)**, and the T cell responses were measured by IFN-γ ELISpot assay. The two-dimensional peptide matrixes were used (as seen in Figure S1), with SFCs > 50 per 10^6 cells was considered as a positive peptide pool, and the candidate peptides were localized within the peptide matrixes. The results were expressed as mean ± SEM.

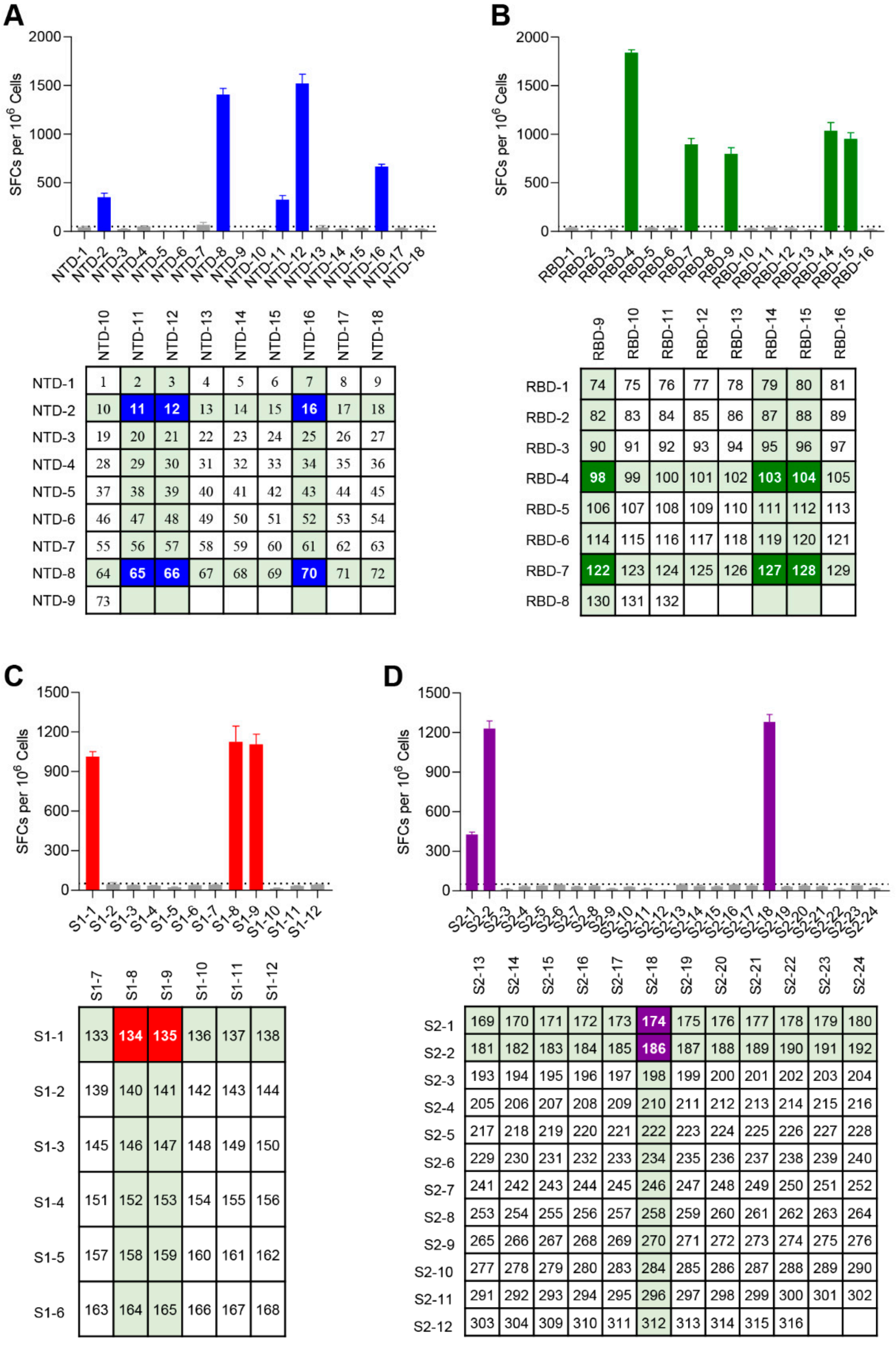

Figure S5. Mapping of Omicron BA.1 Spike specific T cell epitopes in C57BL/6 mice. C57BL/6 mice (n=6 per group) were injected with 5 × 10^8 VP of Ad5-Spike-BA.1. At 2 weeks after vaccination, the splenocytes were prepared, stimulated with peptide pools of Omicron NTD **(A)**, RBD **(B)**, S1 **(C)**, S2 **(D)**, and the T cell responses were measured by IFN-γ ELISpot assay. The two-dimensional peptide matrixes were used (as seen in Figure S1), with SFCs > 50 per 10^6 cells was considered as a positive peptide pool, and the candidate peptides were localized within the peptide matrixes. The results were expressed as mean ± SEM.

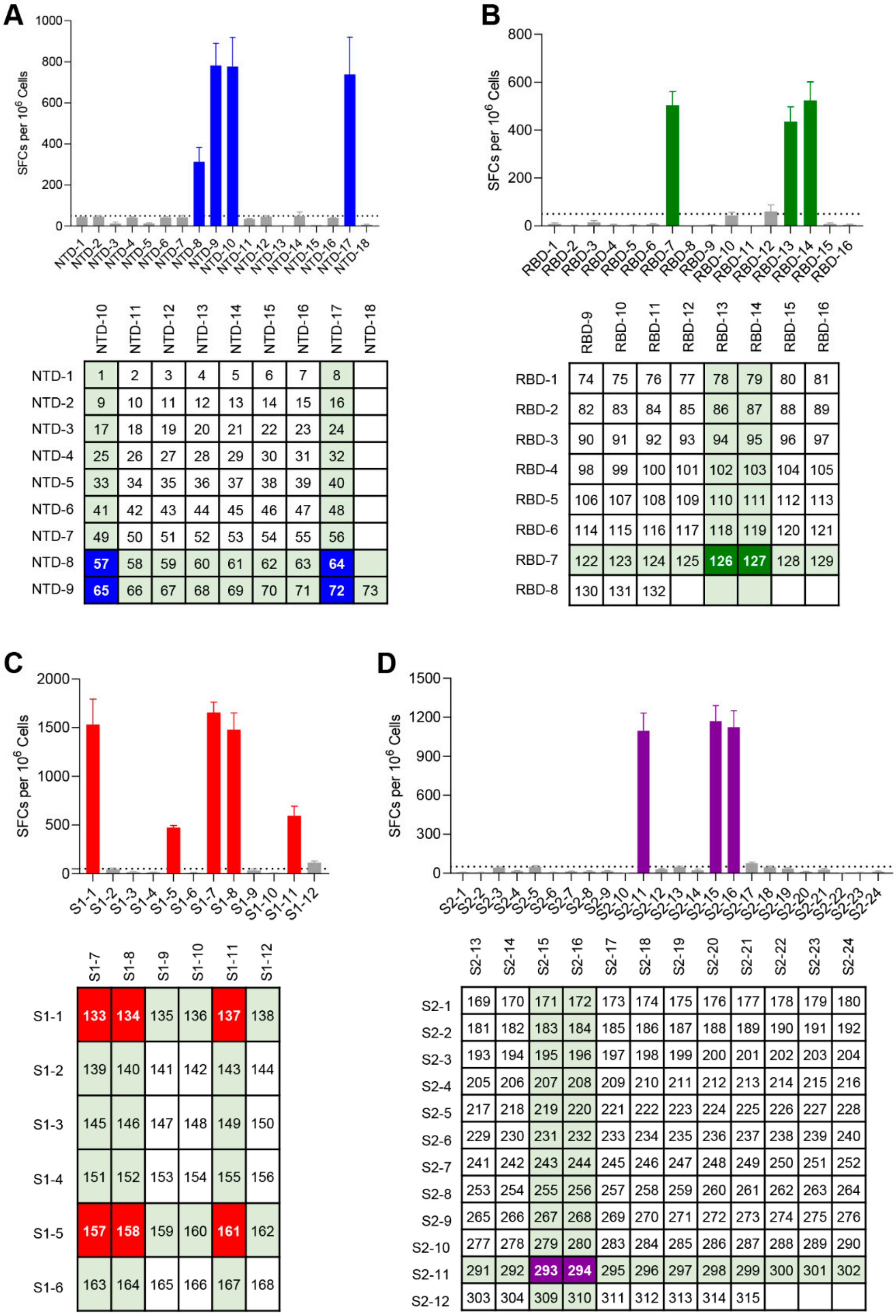

Figure S6. Representative flow cytometric plots of the responsive peptides identified in BALB/c mice. To verify either MHCI or MHCII presented the identified peptides, the responsive peptides in the ELISpot assay were further undergone an intracellular cytokine staining assay, the $CD8^+$ (MHCI) and $CD4^+$ (MHCII) T cell responses of those peptides were determined. The representative flow cytometric plots of the identified peptides in WT Spike **(A)** and Omicron Spike **(B)** were shown.

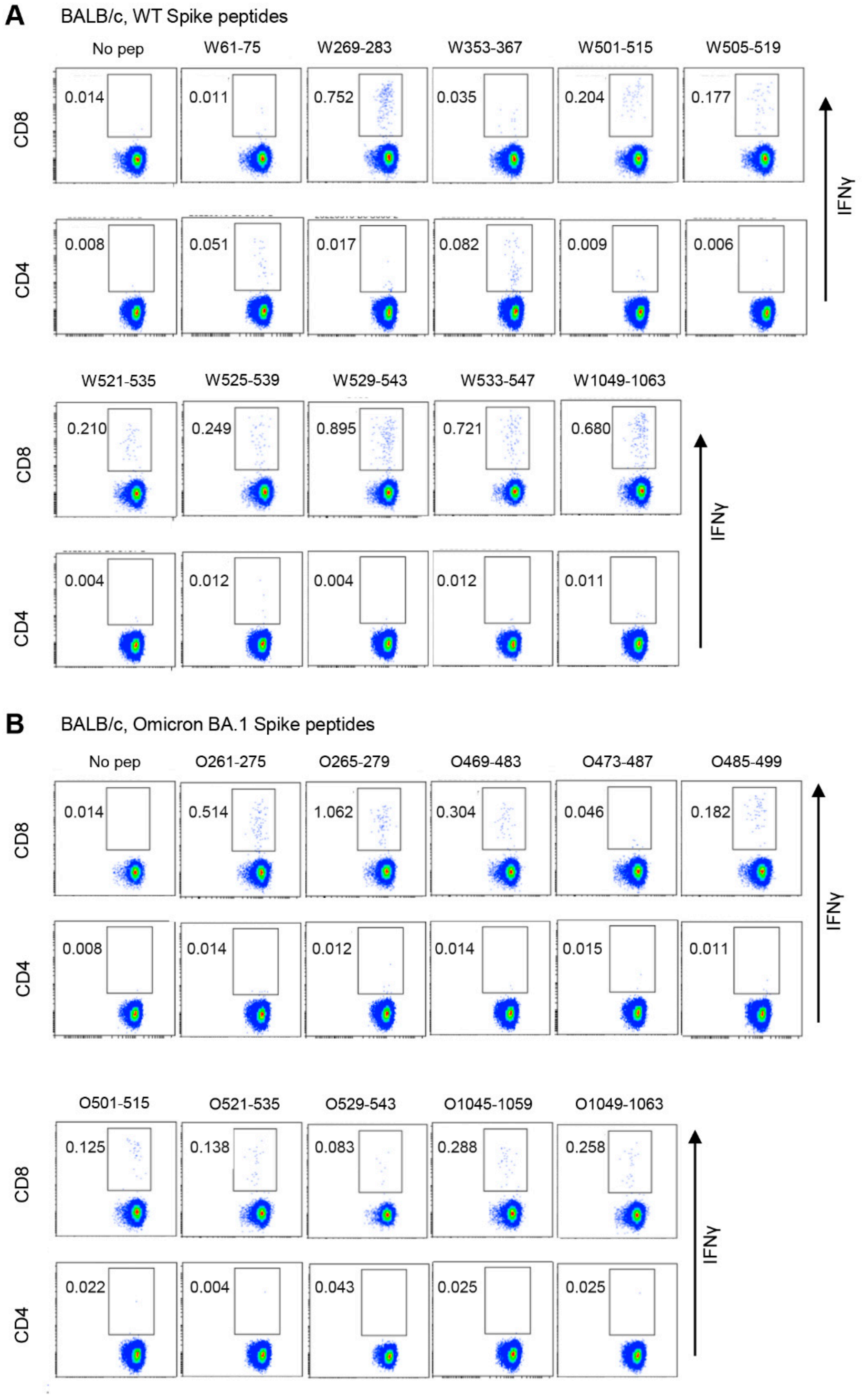

Figure S7. Representative flow cytometric plots of the responsive peptides identified in C57/BL6 mice. With the same strategy in Figure S6, the CD8+ (MHCI) and CD4+ (MHCII) T cell responses of the identified H-2d specific peptides were determined by an intracellular cytokine staining. The representative flow cytometric plots of the identified peptides in WT Spike **(A)** and Omicron Spike **(B)** were shown.

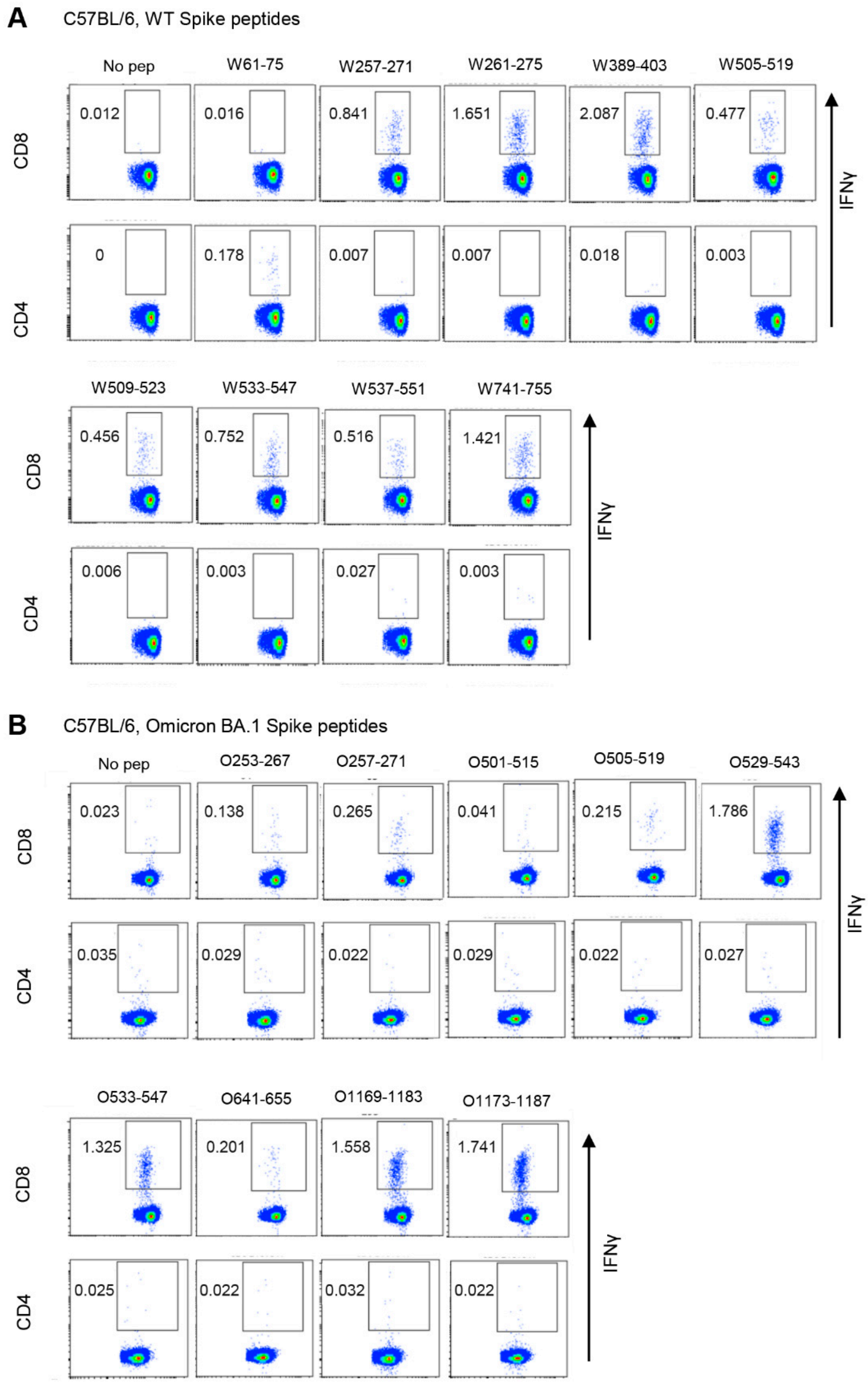

Figure S8. Identification of the exact CD8⁺ T cell epitopes in BALB/c mice. BALB/c mice (n=3 per group) were vaccinated withAd5-Spike-BA.1 or Ad5-Spike-BA.1, the splenocytes were prepared at 2 weeks after vaccination and stimulated with the responsive peptides or the corresponding truncated 9-mer peptides in WT Spike **(A)** and Omicron Spike **(B)** to assess T cell responses by IFN-γ ELISpot. The identified truncated epitopes are labeled with #. All results were expressed as mean ± SEM.

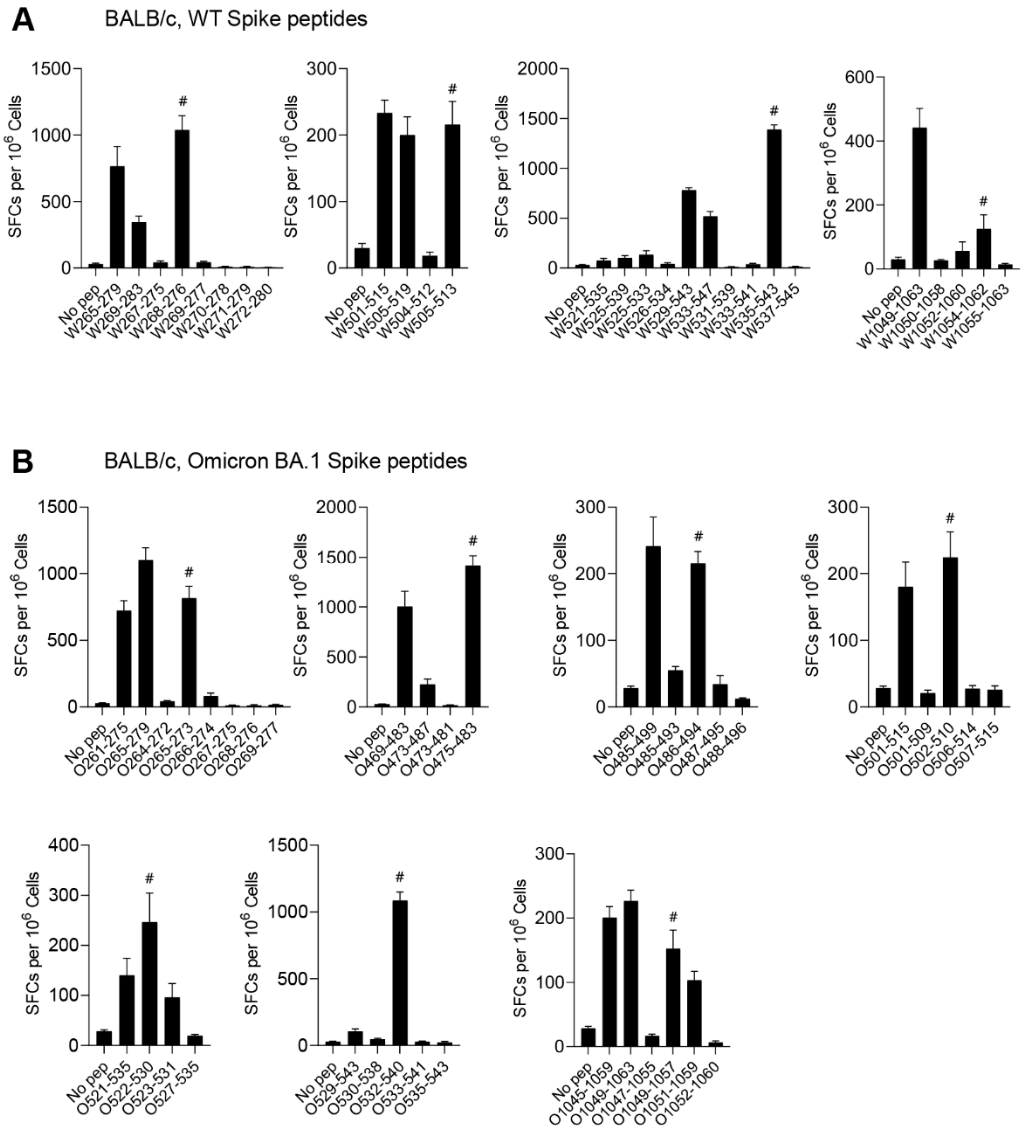

Figure S9. Identification of the exact CD8⁺ T cell epitopes in C57BL/6 mice. C57BL/6 mice (n=3 per group) were vaccinated withAd5-Spike-BA.1 or Ad5-Spike-BA.1, the splenocytes were prepared at 2 weeks after vaccination and stimulated with responsive peptides or the corresponding truncated 9-mer peptides in WT Spike **(A)** and Omicron Spike **(B)** to assess T cell responses by IFN-γ ELISpot. The identified truncated epitopes are labeled with #. All results were expressed as mean ± SEM.

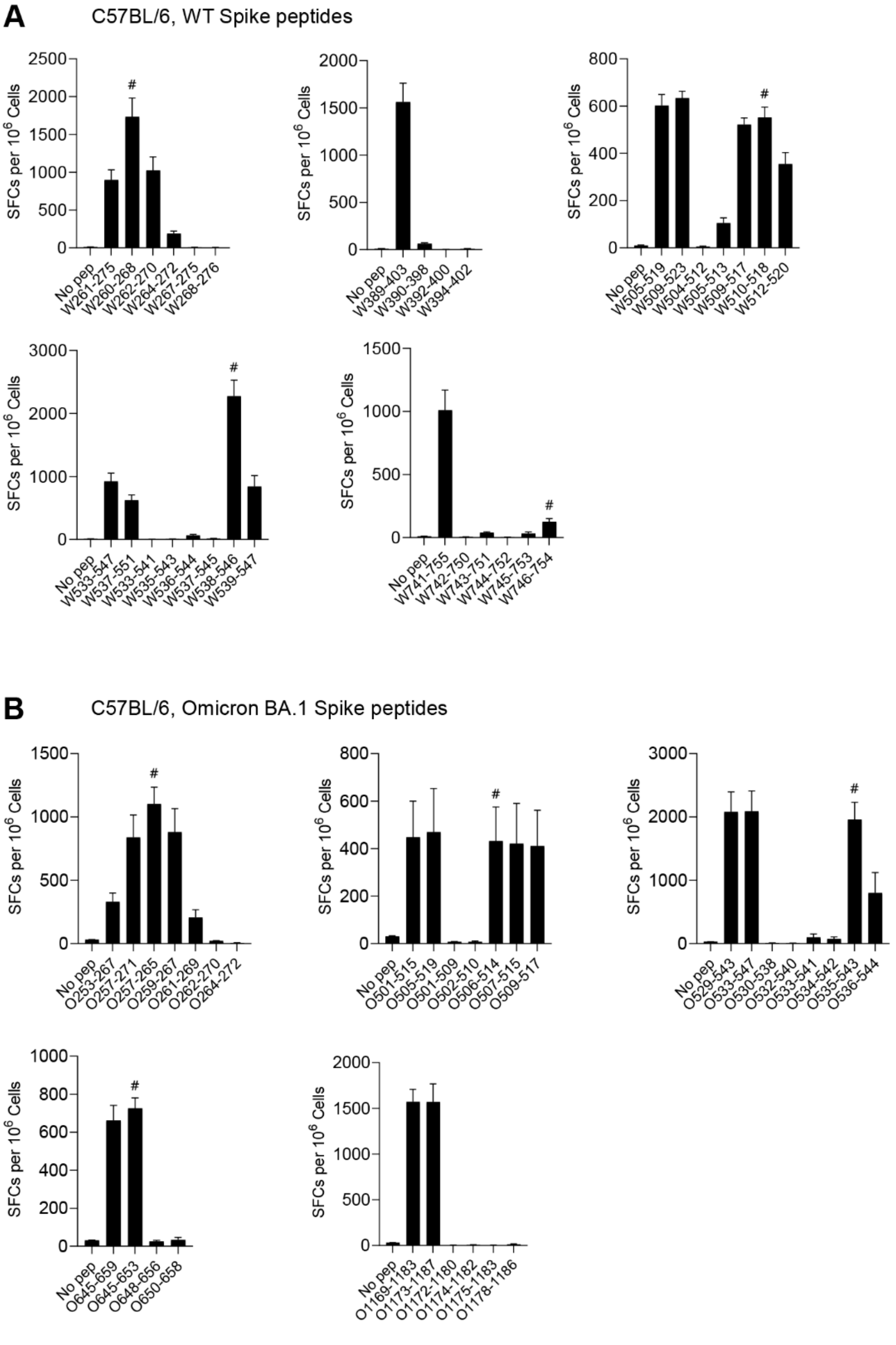

Supplement: Supplementary file 1 [file viruses-15-00763-s001.zip › viruses-2253836-supplementary.pdf]
